# Allogenic Achilles-tendon–bone grafts enable more complete restoration of the native direct enthesis structure in the repair of chronic massive rotator cuff tears

**DOI:** 10.3389/fbioe.2025.1684811

**Published:** 2025-12-02

**Authors:** Fengyuan Zhao, Xinrui Fu, Jiahao Zhang, Lin Lin, Yu Mei, Yu Yin, Jianquan Wang, Hui Yan

**Affiliations:** Department of Sports Medicine, Peking University Third Hospital, Institute of Sports Medicine of Peking University, Beijing Key Laboratory of Sports Injuries, Beijing, China

**Keywords:** massive rotator cuff tear, rotator cuff repair, tendon–bone healing, allograft, bridge repair

## Abstract

Massive irreparable rotator cuff tears are difficult to restore when the tendon quality is poor, and the tendon retraction prevents complete repair. In such cases, tendon allograft bridging can restore continuity but cannot replicate the native tendon–bone interface. In this study, we evaluated an Achilles-tendon–bone block allograft (BTA) for anatomic tendon–bone interface reconstruction in a rabbit model of chronic massive rotator cuff tear. Thirty-six rabbits underwent bilateral infraspinatus tendon detachment, followed by repair after 3 weeks using direct suture (DS), tendon allograft without bone block (TA), or BTA. At 8 and 16 weeks, we assessed the magnetic-resonance-imaging-based tendon maturation (signal-to-noise quotient (SNQ)), micro-computed-tomography-based bone volume fraction (BV/TV) and histology, immunohistochemistry (COL I, II, X), and biomechanical-testing-based healing. The BTA group showed superior tendon continuity, significantly lower SNQ, and higher BV/TV than the DS and TA groups (*p* < 0.05) at both timepoints. The histological examination demonstrated denser collagen fibers, greater fibrocartilage formation, and complete bone–bone fusion in BTA. The immunohistochemical assessment revealed higher COL II and COL X expression, indicating advanced fibrocartilage maturation and mineralization. At 16 weeks, the BTA group achieved the highest ultimate load to failure (113.45 ± 14.45 N) and stiffness (19.65 ± 3.41 N/mm) values, exceeding those of the TA and DS groups (*p* < 0.05). These results indicate that the Achilles-tendon–bone block allograft bridge effectively reconstructs the layered tendon–bone interface, promotes osteointegration and fibrocartilage regeneration, and enhances biomechanical strength, all of which support its potential as a translational option for functional enthesis reconstruction in massive rotator cuff tear repair.

## Introduction

Rotator cuff tears are considered “massive” if they are wider than 5 cm or involve at least two tendons (Jeong et al., 2020). A massive rotator cuff tear usually requires surgical treatment, where surgeons aim to fully repair the rotator cuff as much as possible to restore its anatomy and function (Wong et al., 2021). However, in some cases, the tendon quality may be poor or the tendon may be severely retracted, which makes complete repair impossible. In the past, Burkhart et al. (1994) proposed the concept of partial rotator cuff repair, which focuses on maximizing the restoration of the rotator cuff. When the supraspinatus muscle cannot be fully repaired, the anterior–posterior force couple balance is restored by reconstructing the posterior and anterior parts of the rotator cuff to reclaim its function. Although partial repair of a rotator cuff tear yields satisfactory short-term clinical outcomes for a massive tear, it has a high retear rate ranging from 13% to 94% at long-term imaging follow-up (Mandaleson, 2021; Ishigaki et al., 2021).

To address the issue of poor long-term outcomes in partial rotator cuff repair, bridge repair surgery with patch has emerged as a new surgical option (Chen et al., 2009). This method offers an anatomic structure that provides biomechanical stability while reducing stress on the rotator cuff, making it a more common surgical approach (Wong et al., 2021). The clinically used bridging grafts include patches prepared from autologous tissues (biceps tendon and fascia lata), allogeneic tissues (GraftJacket, fascia lata, and acellular dermal matrix), xenogeneic tissues (Zimmer patch and Conexa), and synthetic materials (Mersilene mesh and Dacron ligament) (Lewington et al., 2017). However, none of these patch materials can fully replicate the layered tendon–bone structure at the insertion point of the rotator cuff.

Recently, Sun et al. (2020) utilized an autologous bone-block-containing Achilles tendon graft to bridge massive chronic rotator cuff tears in a rat model; this approach demonstrated promising histological integration and restoration of the native tendon–bone interface (TBI). However, harvesting the Achilles tendon as an autograft is not feasible in clinical settings owing to its essential physiological role in lower limb function, which limits the applicability of the approach in human rotator cuff reconstruction. Moreover, the study exclusively compared bone-block-containing autologous Achilles tendon bridging grafts with direct suture (DS) repair by focusing on the histological and biomechanical outcomes. Consequently, it remains inconclusive whether the observed advantages were predominantly attributable to the presence of the bone block or to the bridging reconstruction technique itself.

Until now, composite Achilles tendon grafts with bone blocks have already been widely used in clinical procedures, such as the anterior cruciate ligament reconstruction, and have shown reliable clinical outcomes (Lee et al., 2021). This raises an important question of whether a bone-block-containing Achilles tendon allograft can be used to reconstruct the stratified TBI in massive rotator cuff tears to achieve better structural and biological integration. Before such a strategy can be translated into clinical practice, well-designed animal studies are essential to evaluate its feasibility and biological performance.

In the present study, we established a chronic massive rotator cuff tear model in rabbits to compare three surgical strategies, namely, allogenic Achilles tendon grafts with and without bone blocks as well as DS repair. The postoperative evaluations included histological analysis, biomechanical testing, and imaging studies, with particular focus on the structural and healing patterns of the TBI.

We hypothesized that the allogenic Achilles tendon graft with bone block would enable better reconstruction of the stratified enthesis and enhance mechanical strength compared to the graft without bone block or direct repair.

## Materials and methods

### Experimental design

**FIGURE 1:**
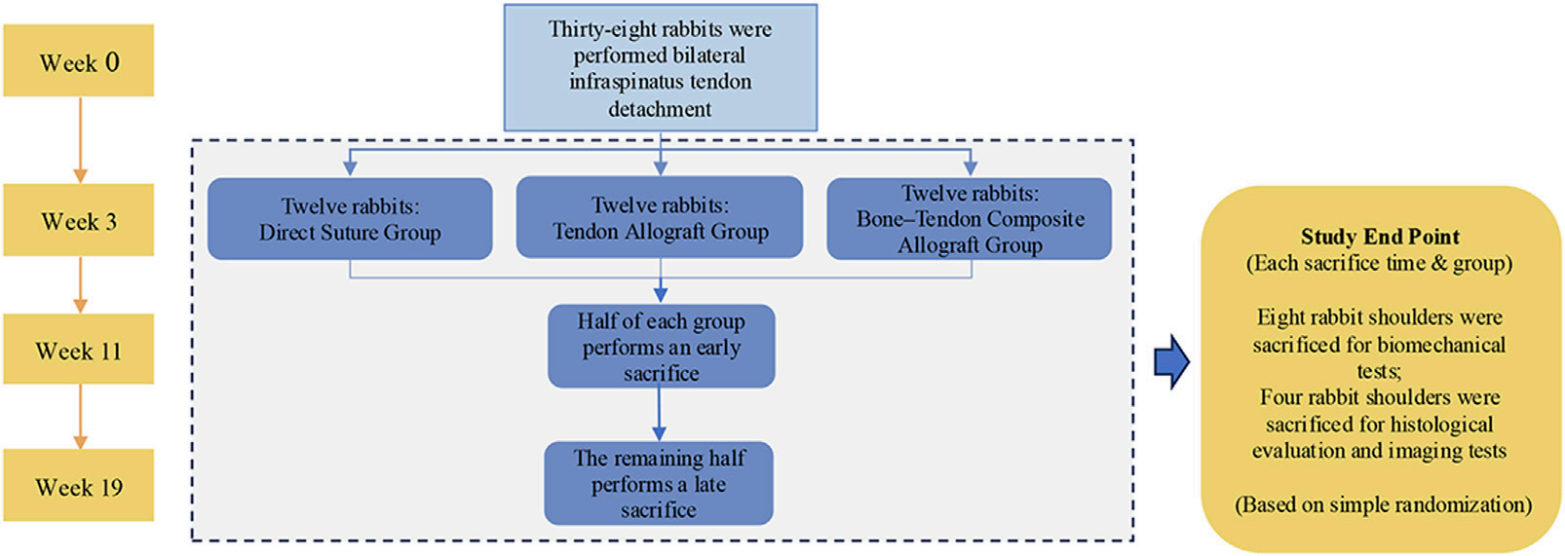
Diagram showing the complete experimental process.

Thirty-six adult male New Zealand white rabbits (24 weeks; mean weight: 3–3.5 kg) were used in this study. The study was approved by the Medical Science Research Ethics Committee of Peking University and was carried out in strict accordance with all regulations. Accordingly, all rabbits underwent bilateral detachment of the infraspinatus tendon, and tendon repairs were performed three weeks later in these animals. The animals randomly underwent either a DS, only a tendon allograft (TA), or a bone–tendon composite allograft (BTA) in each shoulder. Eighteen rabbits were each sacrificed at 8 and 16 weeks after these repairs; twelve of the eighteen rabbits sacrificed at each timepoint were used for the biomechanical tests, while the remaining six were used for histological evaluations and imaging tests. The detailed information regarding the procedures is listed in Figure 1.

### Tendon graft harvesting

The Achilles tendon grafts used in this study were sourced from adult male rabbits used in prior knee experiments. The procurement procedure involved making a 2-cm incision at the posterior aspect of the ankle joint to access the Achilles-tendon–bone interface. For the composite graft group, both the tendon and a bone block were harvested while ensuring that approximately 4 mm of the bone and 6 mm of the tendon were preserved. For the tendon-only graft group, a 1-cm segment of the tendon was extracted from near the calcaneal tuberosity. After thoroughly rinsing with phosphate-buffered saline (PBS), all composite and pure tendon graft samples were stored at −80 °C.

### Surgical technique

**FIGURE 2:**
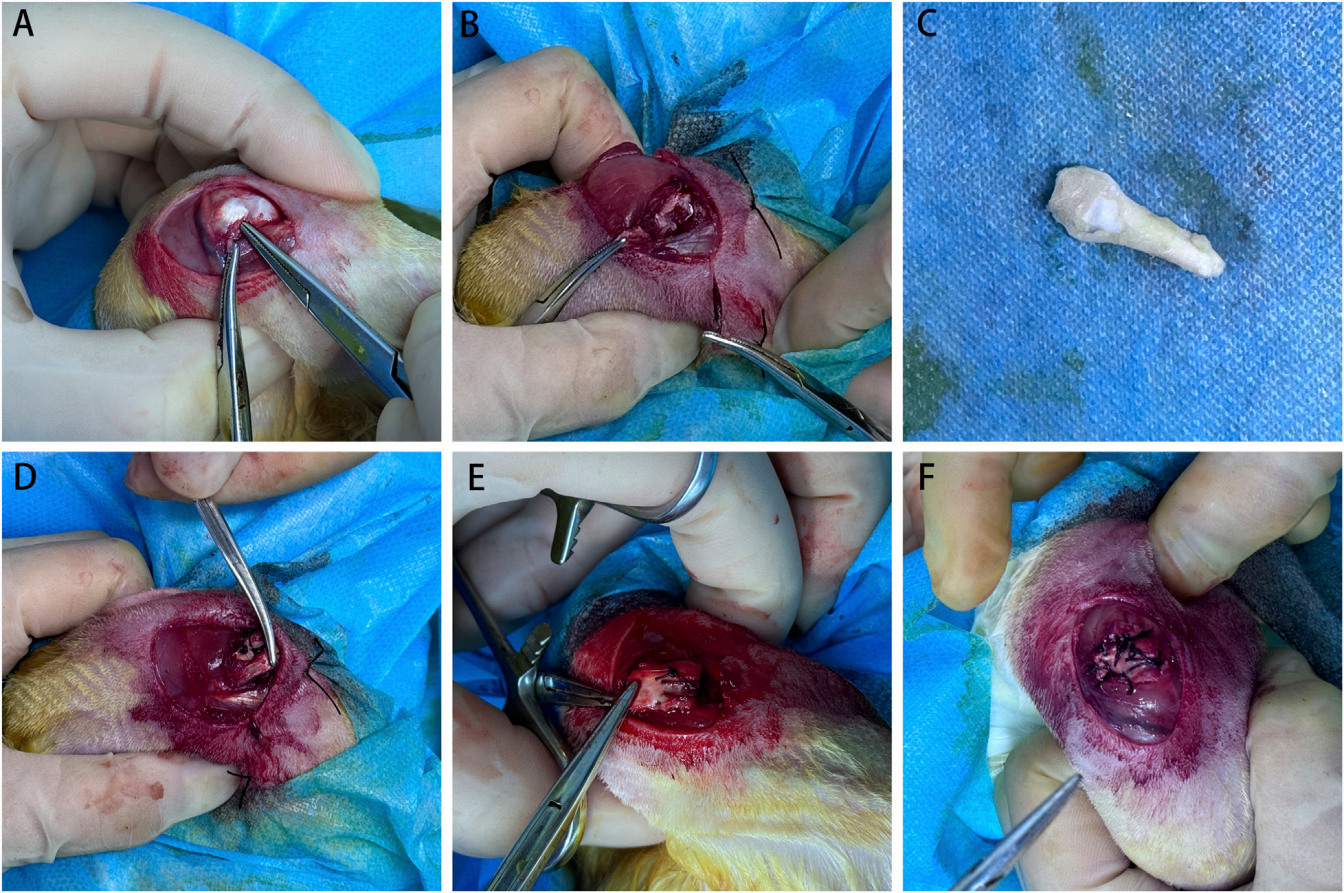
Surgical procedure in the bone–tendon composite graft group. **(A)** During the first surgery, the infraspinatus tendon was located and cut at the tendon–bone junction. **(B)** After the incision, retraction of the infraspinatus tendon was observed. **(C)** Achilles tendon and calcaneus bone composite graft. **(D)** Three months later, during the second surgery, the bone–tendon composite allograft was sutured to the retracted infraspinatus tendon. **(E)** The bone block of the graft was sutured to the greater tuberosity insertion of the infraspinatus tendon through transosseous suturing. **(F)** Finally, the infraspinatus tendon was repaired.

Each animal underwent two surgical procedures. Initially, we performed a bilateral full-thickness detachment of the infraspinatus tendon from the greater tuberosity. Then, a second surgery was conducted three weeks later to reattach the infraspinatus tendon. The repair methods for the rotator cuff tears included bridging repair with the BTA (Figure 2), bridging repair with the TA, and DS repair. The allocation of these repair techniques was randomized for each joint.

For the procedures, the rabbits were first anesthetized via isoflurane inhalation (1%–3% in oxygen; induction at 3% for approximately 2–3 min, maintenance at 1%–2%) delivered via a nose cone at an oxygen flow rate of 1–2 L/min and placed on sterile drapes with the shoulder positioned in flexion and abduction. Next, a longitudinal incision was made on the lateral shoulder, and the muscle was split to expose the retracted tendon. Then, the cortical bone at the tendon insertion site was scraped using a surgical blade to allow exudation of the bone marrow tissue. In the DS group, the infraspinatus tendon was reattached to the footprint using the transosseous suture technique by employing two 3-0 Vicryl plus sutures (VCP772D; Ethicon); here, none of the retracted tendons covered the actual footprint but were partially repaired to the bone as much as possible. In both the BTA and TA groups, the graft footprints were reattached using the same transosseous technique with two 3-0 Vicryl plus sutures. The tendon side of the graft was then adjusted for proper tension and connected to the leading edge of the torn rotator cuff using a modified Kessler stitch with 3-0 Vicryl plus sutures.

Following surgery, the muscles were sutured, and the wound was closed with interrupted sutures. The postoperative care included providing the rabbits with sufficient cage space, food, and water, along with a 7-day course of penicillin G (400,000 U) to prevent infections.

### Magnetic resonance imaging (MRI)

The rabbits were euthanized at 8 and 16 weeks after surgery under deep isoflurane anesthesia (3% for induction, 1%–2% for maintenance in oxygen at 1–2 L/min) administered via a nose cone, followed by intravenous administration of potassium chloride (2 mmol/kg) through the marginal ear vein, in accordance with the AVMA (American Veterinary Medical Association), (2020). Death was confirmed by the absence of a heartbeat and respiration as well as fixed and dilated pupils. The shoulders were then collected for examination, and MRI (3.0 T Omega; United Imaging) was performed to evaluate the maturation of the infraspinatus tendon.

The tendon maturation was quantitatively assessed using the signal-to-noise quotient (SNQ) near the insertion site. Owing to anatomical differences, different representative slices were selected to demonstrate the MRI findings at the TBI. The SNQ was determined using the formula: (signal intensity of the repaired infraspinatus tendon – signal intensity of the native subscapularis tendon)/signal intensity of the background. The signal intensity was measured from a region of interest close to the insertion of the repaired infraspinatus tendon. The background region was located approximately 3 cm behind the infraspinatus tendon, while the native subscapularis tendon was evaluated at the TBI. Two independent investigators performed these measurements, and the average value was used.

### Micro-computed tomography (micro-CT)

The tissues of interest in this study, including the humerus and the infraspinatus tendon attached to the greater tubercle, were excised from the shoulder joint. The specimens were then fixed in 10% neutral-buffered formalin for 72 h. Micro-CT (Xradia 410 Versa; Zeiss) was then used to assess the new bone formation at the TBI under the following scanning specifications: 80 kV, 450 mA, and 0.045-mm effective pixel size.

After scanning, a threshold of 350 Hounsfield units was applied to differentiate the bone voxels of each specimen. Thereafter, 3D reconstructed images were generated, and a 10 mm × 10 mm × 5 mm region of interest was positioned at the TBI. The bone volume fraction (BV/TV) was then calculated for this region of interest using 3D visualization software (Siemens, Inveon, Workplace). The accuracy of this calculation was set to two decimal places. Two independent investigators performed these measurements, and the average value was used.

### Conventional histological assessment

After micro-CT scanning, the specimens were demineralized by immersion in a 20% solution of EDTA (pH 7.3) for 2 weeks. Following this, the specimens were carefully dissected along the sutured infraspinatus tendon to expose the tendon–bone healing junction. Next, the tendon–bone healing junction sections were embedded in paraffin and sliced into 5-µm-thick sections. To assess the microstructure of the tendon enthesis, the sections were stained with hematoxylin and eosin (HE; Solarbio) for general observation of the tendon–bone healing junction and evaluation of the inflammatory responses, safranin O/fast green (SO/FG; Solarbio) and toluidine blue (TB) staining for detection of fibrocartilage regeneration, Masson’s trichrome (MT; Solarbio) for assessing the total collagen content, and sirius red (SR; G-CLONE) for analyzing the composition and semiquantitative assessment of collagen at the tendon–bone healing junction.

For semiquantitative analysis of the MT- and SO-stained sections, the entire interface area as well as areas with blue- or red-positive staining were outlined manually and measured separately using ImageJ software (National Institutes of Health). The ratio of the red-positive area to the total interface area was then calculated with an accuracy of up to one decimal place. The calculations were carried out by two independent pathologists, and the average value was taken as the result (Zhao et al., 2019).

The sections stained with SR were examined under a polarized light microscope (TCS SP8 STED; Leica), where type I collagen (COL I) exhibited strong yellow–red birefringence, whereas type III collagen (COL III) showed faint green under the polarized light. The semiquantitative analyses were performed using ImageJ software, as described in a previous study (Zhao et al., 2014).

### Immunohistochemistry

Collagen types I, II, and X (COL I, II, and X) were detected using immunohistochemistry. Briefly, the sections were incubated with 3% H_2_O_2_ (Lircon) for 15 min to block endogenous peroxidase activity. After rinsing with PBS (Boster Bio), the sections were treated with pepsin solution (ZSGB-Bio) for 1 h to unmask the antigens. The sections were then incubated with goat serum solution (Boster Bio) for 1 h to block non-specific binding. Then, anti-COL I (1:500; Arigo), anti-COL II (1:500; Abcam), and anti-COL X (1:500; Abcam) primary antibodies were applied to the sections and incubated for 60 min at room temperature. Thereafter, the sections were incubated with the corresponding secondary antibodies for 60 min at room temperature. After washing with PBS, a 3,3′-diaminobenzidine (ZSGB-Bio) reaction was performed, and the sections were counterstained with hematoxylin (Solarbio) to reveal the nuclei. The semiquantitative analyses of COL I, II, and X were performed by measuring the percentages of positively stained areas in the defect areas using Image Pro-Plus software.

### Biomechanical testing

**FIGURE 3:**
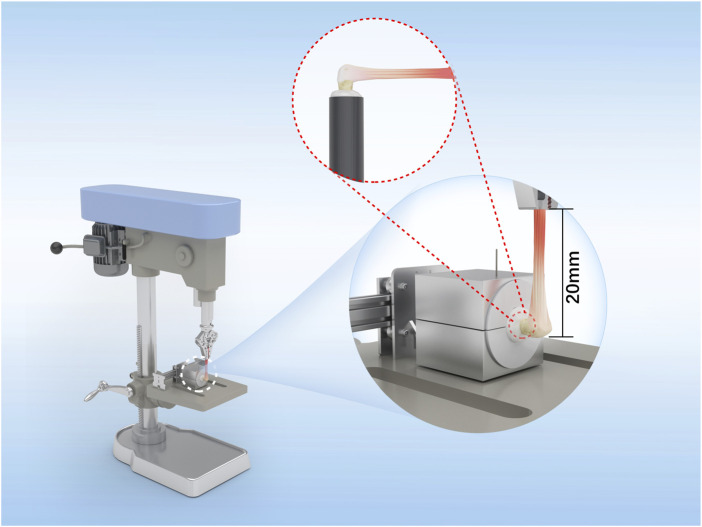
Schematic illustration of the biomechanical test setup. The rabbit humerus was embedded in a polyvinyl chloride (PVC) cylinder and fixed with plaster before mounting on the testing base. The tendon end was secured in a pneumatic grip to prevent slippage during loading. The angle between the humeral shaft and tendon was maintained at 90°, corresponding to the physiological pull direction of the infraspinatus. The initial gauge length (L_0_), defined as the distance from the humeral fixation site to the grip, was set to 20 mm.

For the biomechanical tests, eight operated shoulders from each group were collected at 8 and 16 weeks. The specimens were stored at −20 °C and thawed to 4 °C before testing. This single freeze–thaw protocol preserves the native mechanical properties and minimizes tissue degradation. The proximal humerus was rigidly fixed to a polyvinyl chloride (PVC) cylinder using plaster to ensure stability. The distal end of the tendon was clamped in a pneumatic grip to provide uniform pressure and prevent slippage during loading. The tendon was tested under tension at 90° to the longitudinal axis of the humerus to ensure alignment with the physiological insertion direction of the rotator cuff (Figure 3); the load was applied along this axis to simulate physiological tensile loading conditions. Before testing, the grip-to-potting distance (or initial gauge length L_0_) was set using a positioning jig and verified with a caliper (L_0_ = 20 ± 1 mm). Uniaxial tension was then applied at a crosshead rate of 5 mm/min, corresponding to a nominal strain rate of approximately 0.25 min^−1^. The displacement was then converted to engineering strain (ε = ΔL/L_0_) for stiffness calculations. The system compliance was minimized through the use of a rigid humeral block and tendon screw grip as well as verified using a rigid dummy specimen prior to testing.

During the testing, two types of graft failure were observed, where the first was at the tendon body and the second was at the tendon–bone healing junction. The failure type was documented for each specimen. For both failure types, the load and elongation at the point of graft rupture were recorded, and the load–elongation curve was generated by plotting elongation along the x-axis and load along the y-axis. The linear segment of this curve was fitted using the least-squares regression model, and the slope estimate was considered to represent the graft stiffness. All measurements were recorded up to an accuracy of two decimal places.

### Statistical analyses

All data were presented as mean ± standard deviation (SD), and the statistical analyses were carried out using SPSS 22.0 software for Windows (Chicago, IL, United States) by considering significance at the *p* < 0.05 level. The *in vitro* experiments were repeated >3 times, and the graft failure types between the groups were compared using Fisher’s exact test. One-way analysis of variance with multiple comparisons was performed for the three data groups in both the *in vitro* and *in vivo* experiments. The exact *n* value in each experimental group was >3.

## Results

### Gross observations

**FIGURE 4:**
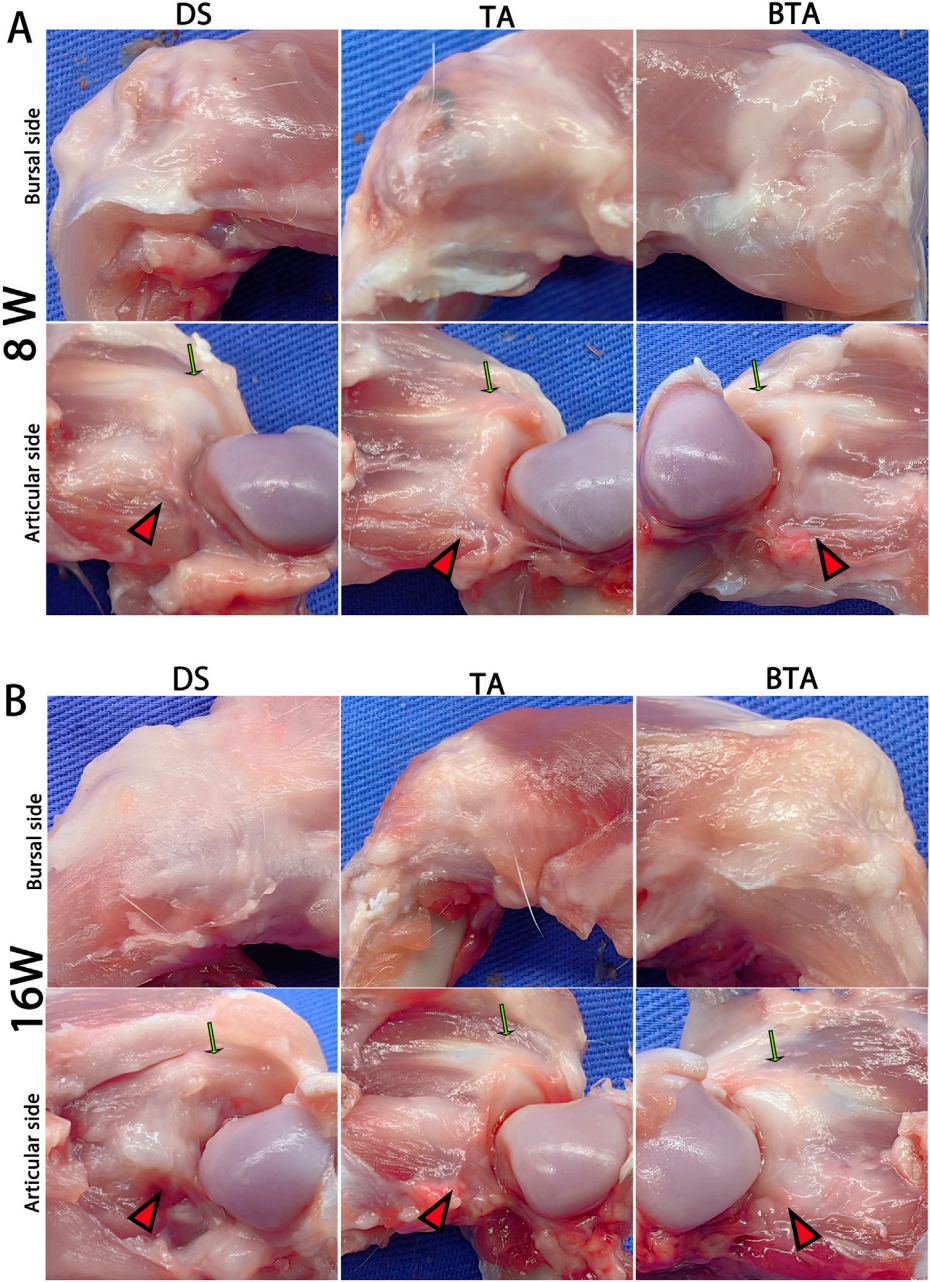
Gross observations on the bursal and articular sides of the specimens at **(A)** 8 and **(B)** 16 weeks postoperatively. The green arrows indicate the intact original supraspinatus tendon, while the red triangles indicate the sutured infraspinatus tendon or graft substance. DS, direct suture group; TA: tendon allograft group; BTA, bone–tendon allograft group.

At 8 and 16 weeks postoperatively, we did not observe any deaths or infections in the rabbits; moreover, the infraspinatus tendon was substantially reattached to the greater tubercle in each group (Figure 4).

The detectable synovium-like tissues covered both the bursal and articular sides of specimens in all three groups at week 8. At this timepoint, the graft was rooted into the greater tubercle in the TA and BTA groups, displaying more oriented and continuous tissues in line with the tensile force of the rotator cuff tendon. By comparison, the sutured infraspinatus tendon was attached to the bone surface without detectable directivity in the DS group (Figure 4A).

At 16 weeks, both the TA and BTA groups had more robust and regular graft substances compared to the sutured tendons in DS group. Notably, the tendon–bone junctions continued to be visible in the DS group. In contrast, the bridging grafts in both the TA and BTA groups resulted in superior continuity and arrangement at the graft–bone junction through the graft rooted into the greater tubercle. Further, the graft substance of the BTA group was intuitively denser and more robust compared to that of the TA group (Figure 4B).

### MRI

**FIGURE 5:**
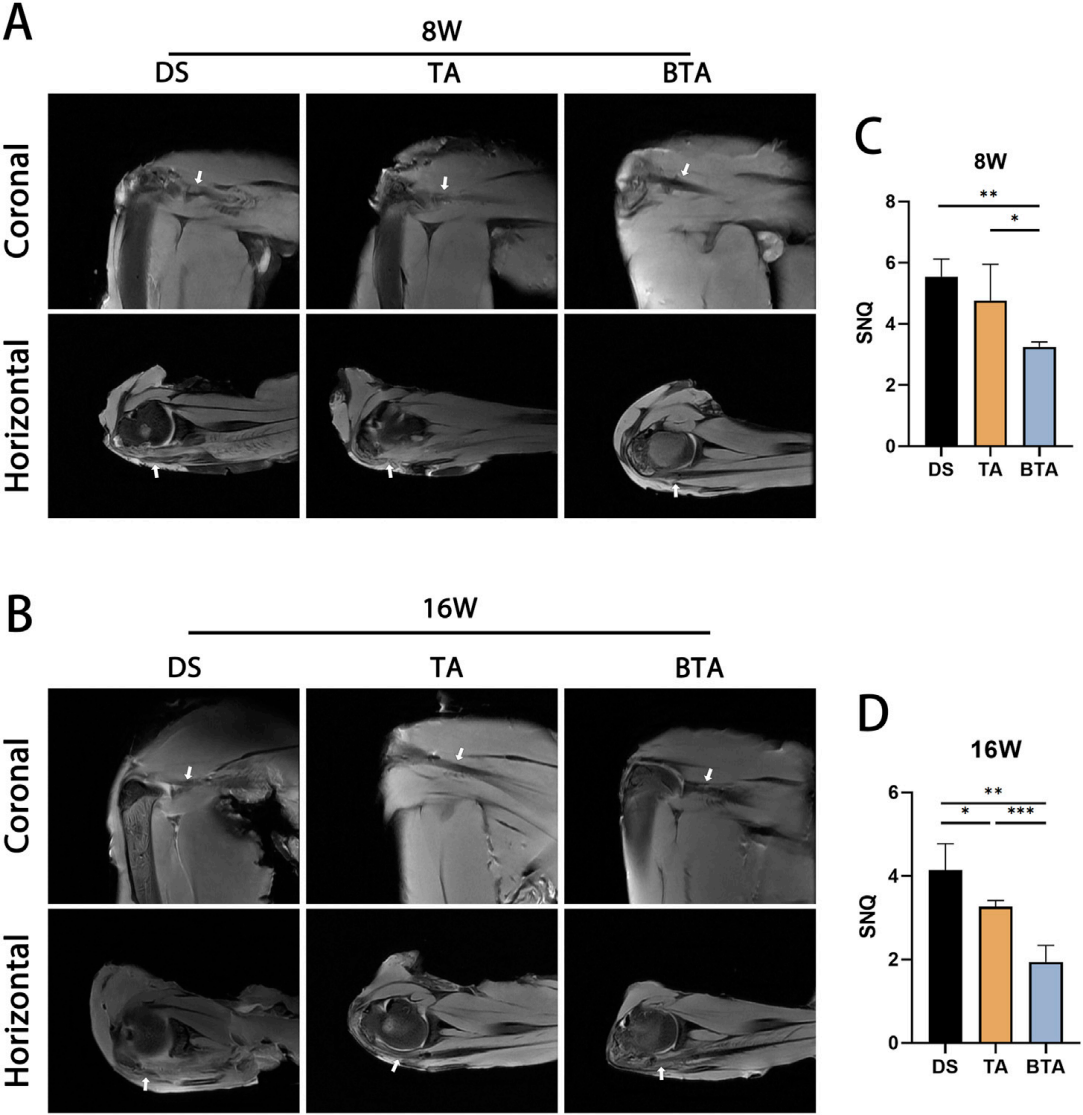
Representative magnetic resonance imaging scans of the direct suture (DS), tendon allograft (TA), and bone–tendon allograft (BTA) groups at **(A)** 8 and **(B)** 16 weeks postoperatively. Different representative slices were selected owing to anatomical variability. The mean signal-to-noise quotient (SNQ) values for the DS, TA, and BTA groups are shown at **(C)** 8 and **(D)** 16 weeks postoperatively. All data are presented as mean ± SD. *p < 0.05; **p < 0.01; ***p < 0.001.

To evaluate the healing of the graft itself as well as the bridging site, we performed MRI of the DS, TA, and BTA groups at 8 and 16 weeks postoperatively to observe the quality of the soft tissue in the TBI (Figure 5). The MRI results revealed that the DS group exhibited a more disorganized tendon structure than the other two groups, potentially owing to the higher suture tension in the coronal plane at 8 weeks. In the horizontal plane, the BTA group exhibited augmented continuity and diminished signal intensity of the infraspinatus tendon at 8 weeks compared to the DS and TA groups. The SNQ of the sutured tendon portion of the BTA group was significantly lower than those of the other two groups (BTA vs. DS: *p* = 0.002; BTA vs. TA: *p* = 0.021), suggesting that the BTA group had less soft tissue edema at 8 weeks compared to the other two groups, which is more advantageous for early healing.

Good tendon continuity was seen in all three groups at 16 weeks. However, the TA graft had lower SNQ than the DS graft (*p* = 0.020), which may be related to the high suture tension at the time of healing in the DS group that could delay healing of the infraspinatus tendon. The BTA group had lower MRI signals at 16 weeks and a significantly lower SNQ than those of the DS and TA groups (BTA vs. DS: *p* < 0.001; BTA vs. TA: *p* = 0.002), suggesting that the BTA group healed more efficiently than the other two groups.

### Micro-CT

**FIGURE 6:**
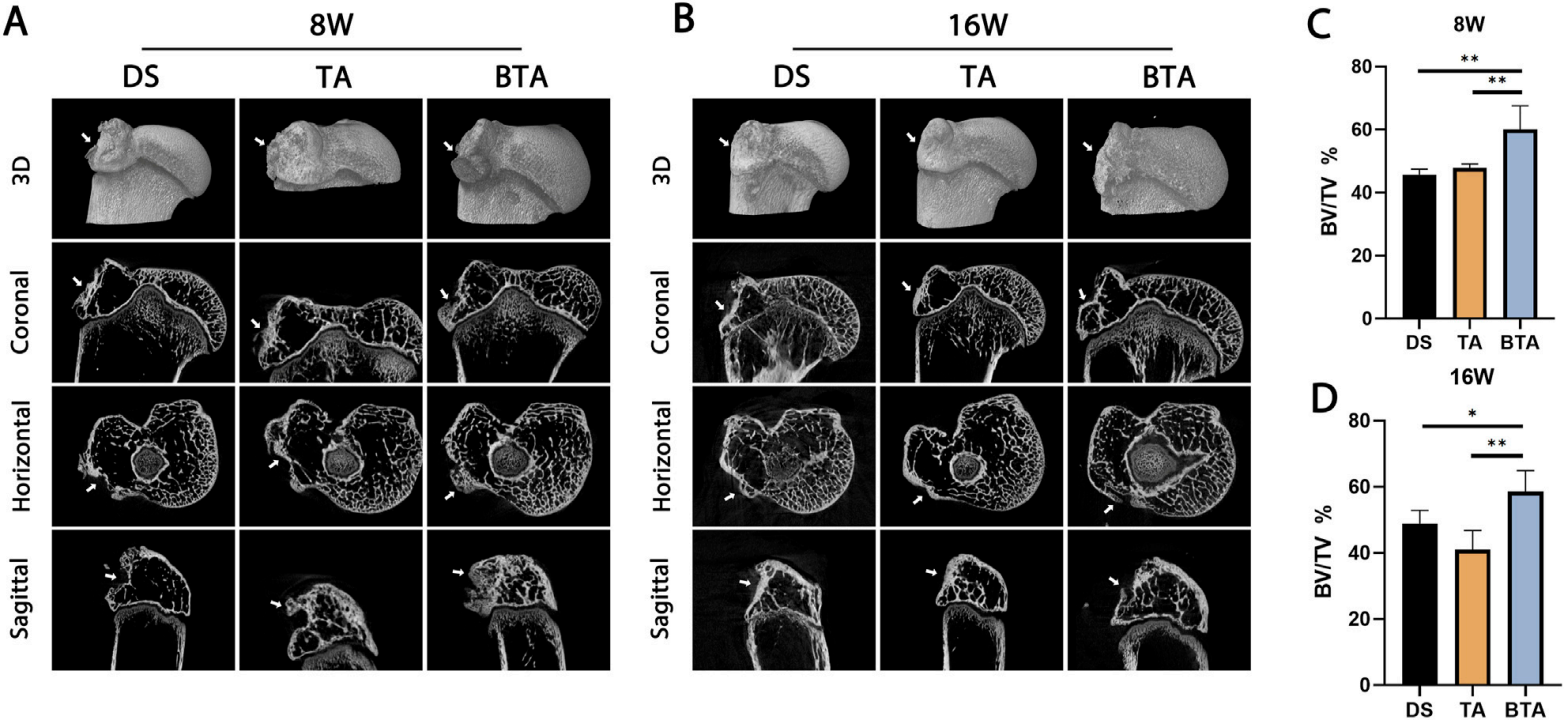
Representative micro-CT scans of the DS, TA, and BTA groups at **(A)** 8 and **(B)** 16 weeks postoperatively. The mean bone volume/total volume (BV/TV) values for the DS, TA, and BTA groups are shown at **(C)** 8 and **(D)** 16 weeks postoperatively. All data are presented as mean ± SD. *p < 0.05; **p < 0.01; ***p < 0.001.

To investigate whether the grafts could facilitate healing at the TBI, we conducted micro-CT imaging to observe the bone growths at the tendon–bone insertion site (Figure 6). All groups displayed evidence of new bone formation at the insertion site at 8 weeks. Among the groups, the allograft bone of the BTA group exhibited fusion with the original bone. At 16 weeks, as tendon–bone healing had progressed, and the TBI structure had become increasingly mature and stable, accompanied by some bone resorption in the bony portions of the new TBI. The formation of the new TBI, characterized by complete fusion of the allograft bone segment with the original bone, was observed in the BTA group. The BV/TV results at both 8 and 16 weeks demonstrated that the BTA group had significantly higher values than the DS and TA groups (8 weeks: DS vs. BTA: *p* = 0.001, TA vs. BTA: *p* = 0.004; 16 weeks: DS vs. BTA: *p* = 0.035, TA vs. BTA: *p* = 0.001). In the BTA group, osseous bone healing occurred, and the bone growth at the TBI was more efficient, resulting in the formation of the new mature and stable TBI.

### Histological assessments

**FIGURE 7:**
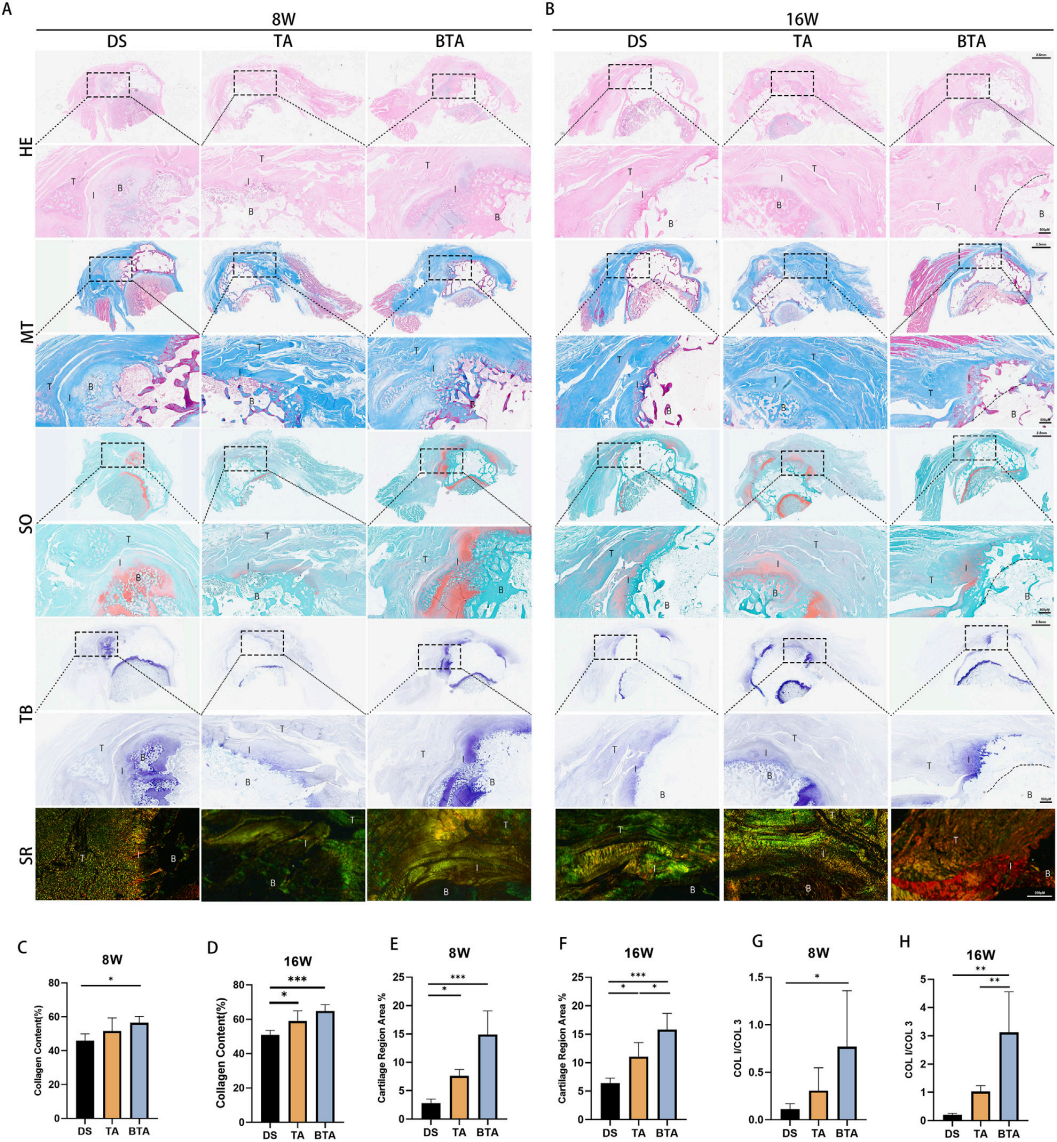
Histological results of the interface regions for the three groups. Representative images of the infraspinatus tendon enthesis stained with hematoxylin and eosin (HE), Masson’s trichrome (MT), safranin O/fast green (SO/FG), toluidine blue (TB), and sirius red (SR) at **(A)** 8 and **(B)** 16 weeks postoperatively. B, bone; I, interface; T, tendon. The mean collagen contents (%) for the DS, TA, and BTA group are shown at **(C)** 8 and **(D)** 16 weeks postoperatively. The mean fibrocartilage region areas (%) for the DS, TA, and BTA groups are shown at **(E)** 8 and **(F)** 16 weeks postoperatively. The mean COL I/COL III values for the DS, TA, and BTA groups are shown at **(G)** 8 and **(H)** 16 weeks postoperatively. All data are presented as mean ± SD. *p < 0.05; **p < 0.01; ***p < 0.001 (one-way analysis of variance).

To explore the healing of the TBI between the groups, we performed histological tests (Figure 7A; Figure 7B). HE staining showed that the DS and TA groups had poor interface continuity and disorganized tendon collagen fibers at 8 weeks. In contrast, the BTA group demonstrated denser tendon collagen fibers and fewer inflammatory cells, likely owing to the preserved native TBI structure in the allografts. After 16 weeks, the TA and BTA groups exhibited lower degrees of inflammatory cell infiltration and more compact collagen fiber structures compared to the DS group, which contributed to enhanced healing at the interface. We observed that there was extensive chondrogenesis between the allograft and native bones in the BTA group at 8 weeks, which was already primed for bone-on-bone healing. The allograft and native bones in the BTA group showed complete healing at 16 weeks.

We found that the BTA group had a wider area of MT staining at 8 weeks than the DS group (*p* = 0.023; Figure 7C). Further, at the late phase of healing by 16 weeks, the TA (*p* = 0.028) and BTA (*p* = 0.001) groups exhibited greater collagen fiber densities than the DS group, suggesting that collagen fibers grow more rapidly and better in the allograft groups, especially the BTA group (Figure 7D). The cartilage area of the TBI in the BTA group was unsurprisingly the largest among the three groups at 8 weeks (BTA vs. DS: *p* < 0.001; BTA vs. TA: *p* = 0.025) since the BTA grafts innately contained a cartilaginous portion of the TBI (Figure 7E). By 16 weeks, the allograft groups had better chondrogenesis than the DS group, with the BTA group also showing better cartilaginous tissue morphology and cartilage staining area (BTA vs. DS: *p* < 0.001; TA vs. DS: *p* = 0.016; BTA vs. TA: *p* = 0.014) (Figure 7F). Through SO and TB staining, we further observed initial bone healing at 8 weeks and complete bone healing at 16 weeks in the BTA group. The 8-week results of SR staining showed that the BTA group had a greater degree of maturation than the other two groups, as indicated by the mean COL I/COL III values (BTA vs. DS: *p* = 0.033) (Figure 7G); at 16 weeks, the tendon tissues at the interface in the BTA group demonstrated greater maturity and had higher COL I expression than in the other two groups (BTA vs. DS: *p* = 0.001; BTA vs. TA: *p* = 0.006), whereas the tendon tissues in the DS and TA groups were more likely to express COL III (Figure 7H). Further, we observed that the COL I/COL III values increased significantly more in the allograft groups, suggesting that the healing process was more efficient in these groups, particularly the BTA group. These histological findings demonstrated that the allografts in the BTA group were more conducive to cellular growth, collagen fiber reorganization, and tissue healing, all of which facilitate faster and better TBI healing.

### Immunohistochemical staining

**FIGURE 8:**
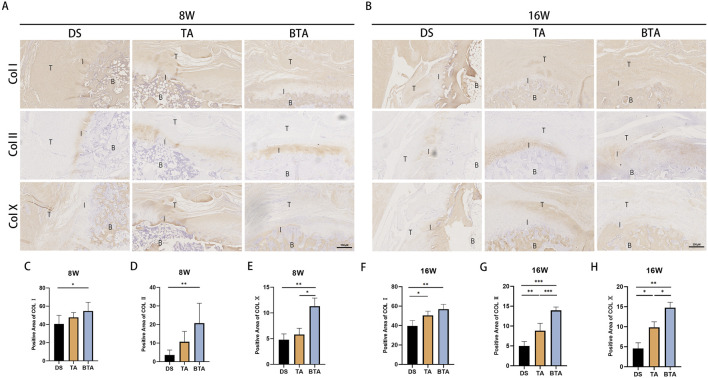
Immunohistological results of the interface regions of the three groups. Representative images of immunohistochemical staining for collagen types I, II, and X (COL I, II, and X, respectively) at **(A)** 8 and **(B)** 16 weeks postoperatively. B, bone; I, interface; T, tendon. **(C–E)** Statistical charts of the positive areas of **(C)** COL I, **(D)** COL II, and **(E)** COL X at 8 weeks postoperatively. **(F–H)** Statistical charts of the positive areas of **(F)** COL I, **(G)** COL II, and **(H)** COL X at 16 weeks postoperatively. All data are presented as mean ± SD. *p < 0.05; **p < 0.01; ***p < 0.001 (one-way analysis of variance). DS, direct suture; TA, tendon allograft; BTA, bone–tendon allograft.

We further examined the fiber compositions of the tendon–bone healing components using immunohistochemical staining for COL Ⅰ, Ⅱ, and Ⅹ for each group at 8 and 16 weeks (Figure 8). The BTA group displayed increased intensity of COL I staining than the DS group (*p* = 0.014) at 8 weeks. We observed denser collagen fibers and higher expression of COL Ⅰ in the BTA (*p* = 0.001) and TA (*p* = 0.014) groups than the DS group at 16 weeks, although there were no significant differences between the BTA and TA groups at either 8 or 16 weeks.

As was evidenced by the results, the BTA group had higher expression of COL Ⅱ in the cartilage region of the TBI than the DS and TA groups owing to the native interface of the graft itself. The BTA group showed a significant difference in the fibrocartilage content compared to the DS group (*p* = 0.008) at 8 weeks. The positive area for COL Ⅱ showed more significant variance in the BTA group than the DS (*p* < 0.001) and TA (*p* < 0.001) groups, and the TA group had greater COL II expression than the DS group (*p* = 0.003) at 16 weeks.

COL Ⅹ is a type of collagen that is mainly found in cartilage and bone. We performed immunohistochemical staining of COL X with the intention of observing fibrocartilage mineralization in the cartilaginous region of the TBI. We observed some COL X expression in the cartilaginous region of the interface at 8 weeks in the BTA group, whereas such expression was largely absent in the DS (*p* = 0.007) and TA (*p* = 0.0016) groups. At 16 weeks, the expression of COL Ⅹ increased in all groups along with mineralization of the cartilage, but the COL Ⅹ expression in the cartilaginous region in the BTA group was much greater than those in the DS (*p* = 0.001) and TA (*p* = 0.033) groups, suggesting that the cartilaginous region of the TBI was most mature in the BTA group. In addition, the TA group had better cartilage mineralization than the DS group at 16 weeks (*p* = 0.024). In conclusion, the TBI healed most effectively in the BTA group in terms of the expression of various types of collagen.

### Biomechanical testing

As shown in Table 1, 8 weeks after the surgery, the failure site was at the TBI for all cases in the DS group. However, there were only five cases for the TA (*p* = 0.028) and four cases for the BTA (*p* = 0.005) groups where the failure site was at the TBI. At week 16, there were no cases of torn grafts at the TBI for both the TA and BTA groups. Furthermore, although there were more interface failure cases in the DS group at week 16, the differences were not significant.

**TABLE 1:** Distribution of the two failure types between the groups.

Follow-up time	Location of failure (tendon–bone or tendon body)		
	DS group (n = 8)	TA group (n = 8)	BTA group (n = 8)
8 weeks	8/0	5/3^a^	4/4^a^
16 weeks	2/6	0/8	0/8

DS group, direct suture of the infraspinatus tendon tears; TA group, tendon allograft was used for the bridge reconstruction of the infraspinatus tendon tears; BTA group, bone-tendon composite allograft was used for the bridge reconstruction of the infraspinatus tendon tears. ^a^ *p* < 0.05 vs. DS group.

**FIGURE 9:**
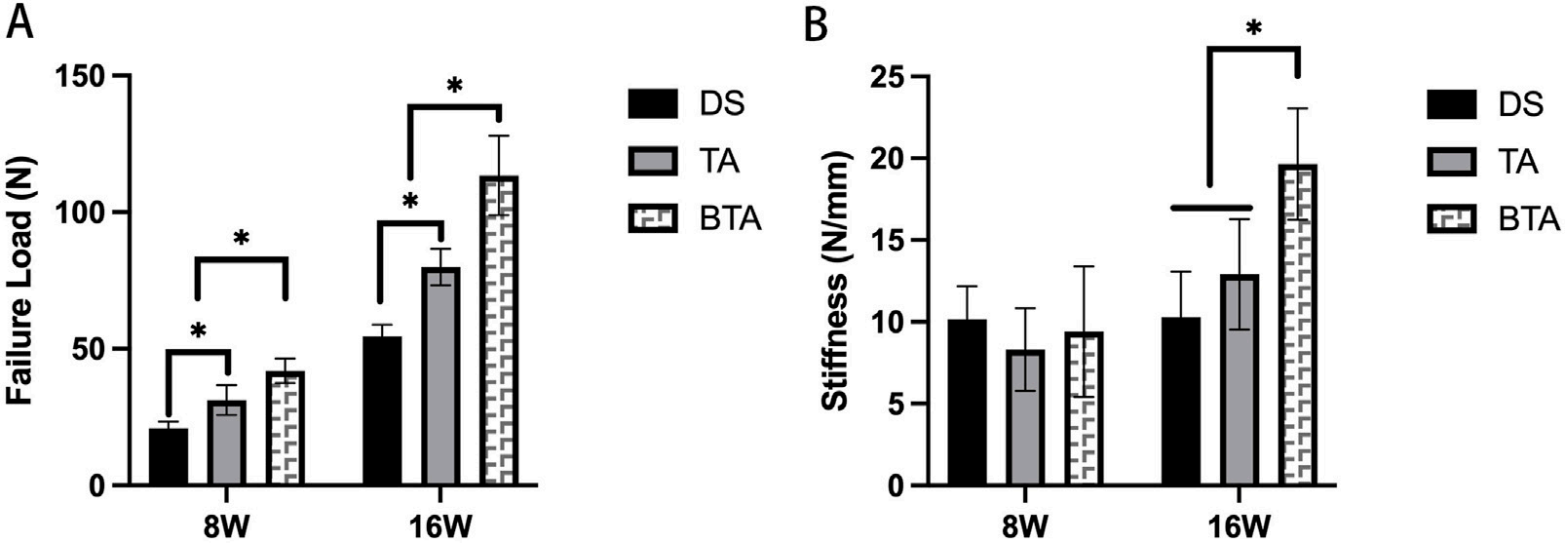
Biomechanical results of the failure load **(A)** and stiffness **(B)** in the three groups. *Significant difference (*p* < 0.05). DS, direct suture of the infraspinatus tendon tears; TA, tendon allograft was used for the bridge reconstruction of the infraspinatus tendon tears; BTA, bone–tendon composite allograft was used for the bridge reconstruction of the infraspinatus tendon tears.

The biomechanical results (Figure 9) show that at week 8, the ultimate load to failure of the specimens in the DS group were significantly lower than those in the TA (*p* = 0.000) and BTA (*p* = 0.000) groups. At this timepoint, the differences in stiffness among the three groups of specimens were not significantly different. At week 16, the ultimate load to failure of the specimens in the TA (*p* = 0.000) and BTA (*p* = 0.000) groups were significantly higher than those in the DS group. Furthermore, the ultimate load to failure of specimens in the BTA group were much higher than 100 N and were statistically different compared to the TA group (*p* = 0.005). At this timepoint, the specimen stiffness values in the BTA group were significantly higher than those in the DS (*p* = 0.000) and TA (*p* = 0.000) groups.

## Discussion

The results of this study show that bridge repair using the BTA or TA is significantly more effective than DS in the treatment of irreparable massive rotator cuff tears. Specifically, the BTA repair method allows effective reconstruction of the native TBI and demonstrates better histological as well as biomechanical healing outcomes than the other two methods.

Allogeneic Achilles tendon grafts have been widely utilized in the reconstruction of tendons and ligaments throughout the body owing to their robustness (Mease et al., 2020). A previous clinical study reported using an allogeneic Achilles tendon graft with a bone block for the bridging repair of massive rotator cuff tears to simultaneously address the greater tuberosity bone defects (Lädermann et al., 2017); the 2-years follow-up study then showed good tendon healing and bony union outcomes (Ho et al., 2022). However, only the clinical scores and imaging results were shown in these works; there is a lack of knowledge regarding the biomechanical strength, bone union status, and histological healing process of the allogeneic Achilles tendon graft with bone block bridging.

Our experiments were designed to fill these gaps. In the present study, we established two control groups, where the first involved an allogeneic Achilles tendon without bone block bridging repair and the second involved the DS method, to determine whether the observed effects are attributable to the bridging technique or the bone block.

We initially compared the TA and DS groups to confirm the advantages of bridge repair over simple suturing. Here, a relatively low number of inflammatory cells and more fibrocartilage tissues were observed in the interface region at 8 weeks in the TA group than the DS group. However, the difference was not significant between the two groups in terms of collagen content, BV/TV value, and SNQ value. We attribute this result to the relatively slow healing rate and poor healing capacity between the tendon and bone (Lui et al., 2010; Xu et al., 2024). Although we utilized the allogeneic Achilles tendon for bridging repair in the TA group, the healing process still occurs between two distinct tissue types (tendon and bone), which cannot restore the layered structure of the rotator cuff insertion site.

At the final timepoint of 16 weeks, the TA group demonstrated relatively superior outcomes in terms of MRI, histology, and biomechanics. At this timepoint, the DS group showed higher SNQ value on the MRI and irregular tendon structures, indicating slower and less stable healing (Figure 4) (Zhang et al., 2019). As a control, the TA group exhibited lower SNQ values owing to the reduced tension at the TBI facilitated by the graft tendon, which promoted better healing. The histological and immunohistochemical results demonstrated that the TBI in the TA group at 16 weeks exhibited more collagen formation and newly generated fibrocartilage (Figure 6 and Figure 7). The presence of chondrocytes and fibrocartilaginous tissues at the TBI is commonly associated with Sharpey’s fiber formation and hence better healing (Lui et al., 2010). The greater collagen and fibrocartilage formation in the TA group at the final timepoint implies better tendon–bone healing results for this group. However, at 16 weeks post-operation, the layered direct insertion structure could not be identified in either the DS or TA group.

Next, we compared the findings from the BTA group with those from the other groups. The BTA group utilizing the tendon–bone allograft showed better repair outcomes than the TA and DS groups at both the early healing and final follow-up stages. This is in accordance with findings of a previous study reported by Smith et al. (2012), who noted the advantages of bone–tendon allografts over the direct repair technique augmented with a human dermis patch (GraftJacket); in contrast to this study, which solely compared the BTA and direct suturing techniques, we incorporated an addition simple tendon bridging group as a control to highlight the value and significance of the bone block. Our micro-CT analyses and histological examinations in the rabbit model of chronic massive rotator cuff tears demonstrated enhanced allograft bone incorporation in the BTA group, which achieves rapid osteointegration and establishes a functional muscle–tendon–bone unit to restore shoulder biomechanics during the early healing phase. COL I, II, and X were also evaluated in our study to better observe the TBI from the perspectives of tendon, hyaline cartilage, fibrocartilage, and finally ossification into the bone (Du et al., 2024). Our results show that compared to the other two groups that healed at the TBI, the BTA group converted healing at the TBI to more rapid bone–bone integration owing to the preexisting natural TBI along with more significant COL II and X expression, which promote healing of chronic massive rotator cuff tears.

Bone repairs can be divided into primary and secondary fracture healing (Loi et al., 2016). In contrast to primary fracture healing observed in autologous bone grafts, the allografts typically undergo secondary fracture healing by a process referred to as endochondral ossification (Thompson et al., 2015). Endochondral ossification is a crucial mechanism for bone healing and is pivotal in facilitating integration between host bone and allograft tissue. During this process, the chondrocytes undergo sequential transformation into osteoblasts and subsequently mature bone cells, thereby orchestrating the bony union and promoting effective bone regeneration (Wolff and Hartmann, 2019). The histological analysis conducted in this study revealed substantial formation of a cartilaginous interface zone between the allograft and host bone at the 8-week timepoint (Figure 6A). This morphological evidence strongly indicates that the allograft material has significant osteoconductive capacity that facilitates bone regeneration (Bostrom and Seigerman, 2005). This specific pattern of endochondral ossification appears to be influenced by not only the origin of the graft material but also the mechanical stability provided by the fixation method (Olate et al., 2019). The histological evaluations demonstrated complete mineralization and advanced bone remodeling at the interface of the allograft and host bone 16 weeks after implantation, which were accompanied by a substantial reduction in inflammatory responses. These histological findings are consistent with those of a previous study reported by Athanasiou et al. (2010), who noted similar patterns of endochondral ossification and tissue integration. Furthermore, the micro-CT analysis demonstrated progressive osseointegration with initial graft–bone interface healing observed at 8 weeks and complete bony union establishment by 16 weeks post-implantation. The histological and micro-CT findings, which revealed distinct patterns of bone–bone integration, were consistent with the biomechanical test results that demonstrated significantly greater maximum load and stiffness values for the BTA group (Figure 9).

Firm attachment of the tendon and greater tuberosity bone at the insertion site provide the requisite foundation for the success of the massive rotator cuff tear repair surgery (Lim et al., 2023; Xu et al., 2022a; Xu et al., 2022b). In recent years, advancements in surgical techniques for rotator cuff tears have supported the clinical outcomes of small- and medium-sized rotator cuff tears with relatively reliable data (Holtedahl et al., 2022). However, massive irreparable rotator cuff tears remain a significant challenge (Kovacevic et al., 2020). Our study demonstrates that the Achilles tendon and bone block allograft bridging technique allows superior histological healing and higher biomechanical strength in the reconstruction of massive irreparable rotator cuff tears. These findings provide a scientific foundation for the clinical applications of this graft for treating massive rotator cuff tears, potentially enhancing the long-term outcomes and success rates of rotator cuff reconstruction procedures. The Achilles tendon and bone block allograft bridging technique thus enables anatomical reconstruction of the layered structure at the rotator cuff insertion site and represents a promising therapeutic approach for future rotator cuff repair procedures.

Given the encouraging findings of this study, we also note that there are some limitations that must be addressed. First, this study is an animal experiment, and the results cannot be directly transferred to humans. Second, the use of non-aged rabbits in our study represents a limitation as it does not accurately reflect the clinical reality that massive rotator cuff tears predominantly occur in the elderly population. Third, our study had a relatively short postoperative follow-up period of 16 weeks, which may be insufficient to fully evaluate the long-term clinical outcomes of the surgical procedure. Fourth, the higher BV/TV ratio observed in the BTA group likely reflects the presence of preexisting bone tissue in the graft, which is an inherent structural feature of this model rather than a direct indication of enhanced biological osteogenesis. Hence, prospective multicenter clinical trials should be conducted in the future to compare the outcomes of the proposed allograft bridging technique with alternative surgical approaches for massive rotator cuff repairs.

## Conclusion

The Achilles tendon and bone block allograft bridging technique suggested herein demonstrates superior capability in terms of reconstruction of the layered structure at the rotator cuff insertion site when repairing massive tears, resulting in enhanced biomechanical strength of the repaired rotator cuff. The improved histological results and biomechanical strength lay the groundwork for future clinical applications of this allograft bridge technique. Nevertheless, further research efforts are needed to determine the complete action mechanism of this method and whether this technique could be applied in clinics. Our findings suggest that the Achilles tendon with bone block allograft could be a favorable graft choice for bridge repair of irreparable massive rotator cuff tears.

## Data Availability

The original contributions presented in the study are included in the article/supplementary material, further inquiries can be directed to the corresponding authors.
